# Exploring New Potential Applications for Hand Exoskeletons: Power Grip to Assist Human Standing

**DOI:** 10.3390/s21010030

**Published:** 2020-12-23

**Authors:** Jorge A. Diez, Victor Santamaria, Moiz I. Khan, José M. Catalán, Nicolas Garcia-Aracil, Sunil K. Agrawal

**Affiliations:** 1 Biomedical Neuroengineering Group, Universidad Miguel Hernández, 03202 Elche, Spain; jcatalan@umh.es (J.M.C.); nicolas.garcia@umh.es (N.G.-A.); 2 Robotics and Rehabilitation Laboratory, Columbia University, New York, NY 10027, USA; vs2578@columbia.edu (V.S.); mikhan@bwh.harvard.edu (M.I.K.); sunil.agrawal@columbia.edu (S.K.A.)

**Keywords:** exoskeletons, rehabilitation robotics, biomechanics, electromyography, upper limb, postural assessment

## Abstract

Hand exoskeleton potential applications reach further than grasping or assistance during manipulation. In this paper, we present a preliminary study of how this technology can be applied in order to improve performance during standing to help the user to keep balance under perturbations. Non-impaired users wearing a hand exoskeleton gripping a hand rail were pushed by a cable-driven robot, so that their standing equilibrium was perturbed. The center of pressure, surface electromyography, and interaction force data were recorded in order to assess the performance of users and their postural strategy. The results showed that users could keep their balance with the same outcomes using their bare hands and the hand exoskeleton. However, when wearing the exoskeleton, a higher muscular activity was registered in hand flexor muscles. This is also supported by the grasping force, which shows that users stretched their hand more than expected when wearing the hand exoskeleton. This paper concludes that it is possible that the lack of tactile feedback could lead to over compensation in the grasping. Therefore, the next studies will aim to check whether this effect can be reversed by training users to wear the exoskeleton.

## 1. Introduction

In the current literature, one can find a wide range of robotic hand exoskeleton devices with different architectures, such as rigid links [1,2], cable driven gloves [3], or soft actuators [4,5]. Furthermore, reviews on hand exoskeletons [6,7] coincide in the applicability of these devices for the rehabilitation or assistance of manipulative skills.

Additionally, multiple studies highlight the role of the hands during other daily tasks beyond dexterous manipulation. For example, many studies have pointed out that the feedback that is provided by a light touch with the fingers can significantly improve the sway of the human center of mass during stance—sensory modulation hypothesis—[8,9,10]. Moreover, researches have shown that the typology of grasping and support can have a significant influence in feedforward and feedback control mechanisms of postural adjustments [11,12]. Hall et al. [11] showed how able-bodied individuals could modulate body and muscular responses while offering diverse kinds of support during one leg stance. In line with this body of literature, adequate motor control of hands seems to be critical, not only during reach-to-grasp or manipulative tasks, but also for postural control purposes. A wide variety of neuromotor disorders, such as stroke or cerebral palsy, present with postural as well as hand control deficits. Stroke survivors develop hemiplegia, a condition that is characterized by muscle paresis, abnormal muscle tone, spasticity, disrupted postural control, and inefficient modulation of grasping forces, among other factors [13,14,15]. In stroke rehabilitation, when the potential for recovery postural control is highly compromised, patients may require specific training in upper limb and reaching strategies in order to overcompensate for the lack of balance deficits during community participation [16]. Therefore, when hand control is highly affected in this population, the use of neuro-technologies, like robotic exoskeletons, to maximize hand functionality and overcompensate postural control deficits during unbalanced conditions would be granted.

In view of these synergies between the hand and human postural control system, the effect of using a hand exoskeleton to assist this supportive gripping may be an interesting research line, which might result in a direct impact in the improvement of the daily living of hand-impaired people. Actually, one can think in several daily situations, in which the postural control enhanced by the hand gripping plays an important role, such as: keeping the balance in public transport during rush hour while texting, avoiding an obstacle or step by holding a handrail, supporting on a cane while transporting an object, etc. These tasks require bi-manual skills, so the robotic assistance of an impaired or weak hand can result in an improvement in the independence of its user.

As a starting point for this research line, we proposed studying the effect that gripping a handrail with a hand exoskeleton has on the postural control of able-bodied subjects (without hand or postural impairments) undergoing controlled external perturbations via motorized-cable driven belts. In this regard, the next set of hypotheses has been formulated:

**Hypothese** **1** **(H1).** *Intuitively, holding a handrail with the hand exoskeleton improves postural performance—stable center of pressure.*

**Hypothese** **2** **(H2).** *Subjects holding a handrail with and without the hand exoskeleton demonstrate similar postural performance.*

**Hypothese** **3** **(H3).** *In this exploratory hypothesis, we test the adjustments of arm muscle patterns with the use of the hand exoskeleton for future applications in patient populations (i.e., stroke).*

Additionally, a Post-Hoc analysis of the recorded data was performed in order to look for feasible correlations between different factor, so new hypotheses can be stated to design further experiments and dig more deeply in the influence of the wearable hand devices in the human control strategies.

## 2. Materials and Methods

### 2.1. Subjects

In this study, eleven subjects have been recruited from the staff of the Mechanical Engineering Department of the Columbia University. All of them without major physical or psychomotor impairments, with an average age of 28 ± 3 years, height (ground to shoulder) of 1.43 ± 0.1 m, and weight of 808 ± 160 N. From this set of eleven subjects, one of them has been removed from the study, due to a loss of data for one of the conditions.

All of the subjects gave their informed consent for inclusion before they participated in the study. The study was conducted in accordance with the Declaration of Helsinki, and the protocol was approved by the Ethics Committee of Miguel Hernandez University with reference number DIS.NGA.01.14-2 and registration number 2017.32.E.OEP.

### 2.2. Experimental Setup

**Figure 1:**
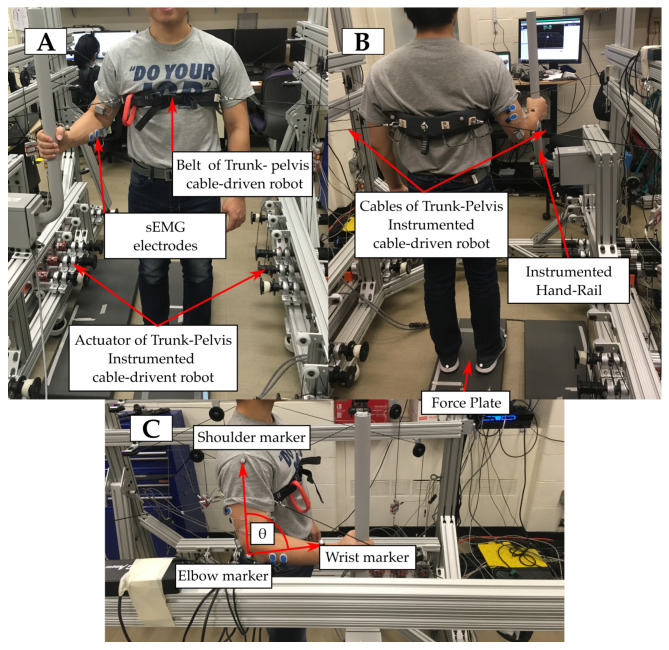
Overview of the experimental setup. (**A**) Frontal view of the setup with a subject in the HR condition, showing the grasping pose. Blue frame shows a detail of the same part in HR + E condition. (**B**) Rear view of the setup shows the placement of the markers that track the position of the end effector of the cable driven robot (belt). (**C**) Lateral view shows the position of the different markers to estimate the angle of the elbow. Indicators of the position of the different sEMG electrodes are distributed across the three pictures.

As shown in Figure 1, the subjects stood on a force sensing plate with their feet 15 cm apart, with their chest position controlled by a Trunk–Pelvis cable driven robot, the Stand-Trainer [17].

To perform this study, three experimental conditions have been stated, and each of them will be performed by all the subjects subsequently, with resting periods of 3 min. between: unsupported (US), hand rail support without exoskeleton (HR), and hand rail support with hand exoskeleton (HR + E). They can be summarized, as follows:

- US: subjects stand on the force sensing plate without any external support. The subjects are asked to try to keep their current pose and position in a natural way, while the robot applies a series of five force perturbations. Subjects are able to take a step if they consider it to prevent falling. The subjects are allowed to recover their initial position and pose before the next perturbation is applied.
- HR: the subjects stand on the same force sensing plate but gripping with their right hand a vertical instrumented hand rail placed at their right side. While gripping the bar, their elbow must rest at an approximate angle of 90${}^{\circ }$ with respect their upper arm and body’s vertical axis. They are asked to keep their current pose in a natural way with the assistance of the hand rail, while the robot applies a series of five force perturbations. They are allowed to take a step if they consider it to prevent falling. The subjects are requested to recover their initial position and pose before the next perturbation is applied.
- HR + E: this condition has the same conditions than HR, but the subjects grip the hand rail with the assistance of a hand exoskeleton [17]. Hand exoskeleton controls the pose of the fingers of the subject and it keeps them wrapped around the hand rail with a stiff position control, so, if the hand of the subjects tries to open, the exoskeleton will apply a force to close it.

All force perturbations are equal within subjects and they consist in a ramp force signal from 0 N to the maximum force that spans a time interval of 1 s, followed by an interval of 0.5 s, in which the maximum force is held. After this force drops to 0 N with a step. The maximum force is different for each subject and it corresponds to 15% of their body weight. The force is always applied in the same direction: perpendicular to the frontal plane in backwards direction.

Regarding the order of the conducted conditions, the condition US has always been performed first (as a baseline of the subject’s performance) and the HR and HR + E order is switched for each subject: so, half of them have performed the sequence US, HR, HR + E, and the rest US, HR + E, HR.

### 2.3. Hand Exoskeleton

**Figure 2:**
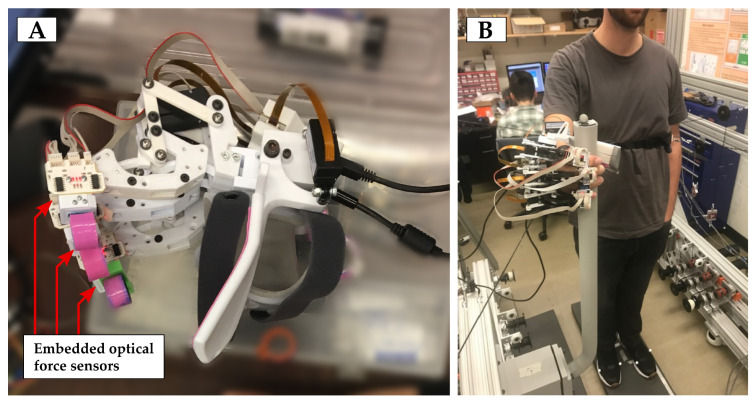
(**A**) Hand exoskeleton used in the experimentation and a detail of the the location of the optical force sensors that are used to measure the interaction force with the hand-rail. (**B**) Subject using the hand exoskeleton to grip the hand-rail in the HR + E condition.

The hand exoskeleton [18] (Figure 2) is an under-actuated robotic device that aimed to assist the user during the grasping of objects that are present in activities of daily living. In particular, it has been designed to grasp cylindrical-shaped objects, such as cups, bottles, or adapted cutlery. The hand exoskeleton has four active degrees of freedom corresponding to: Index finger flexion-extension, middle finger flexion-extension, ring and little fingers flexion-extension, and thumb flexion-extension in opposition. The three degrees of freedom that correspond to the fingers are driven by three equal finger modules that use a bar linkage that is commanded by a linear actuator in order to couple the movement of the different phalanxes and induce the flexion or extension of the finger. As for the thumb degree of freedom, it has been decided not to directly apply a constrained motion to the thumb of the user, since it is expected to have users with uneven thumb abnormal configurations, due to different diseases. Therefore, the thumb module consists of a lever mechanism that replaces the opposition function of the actual thumb without requiring it to move in a concrete pattern, but offering a comfortable support to the user’s thumb. All of the modules have a position feedback signal, which allows for the low level controller to perform a PID position control of the user’s finger pose. Additionally, all finger modules but the thumb are equipped with an embedded optical force sensor [19], which can measure the interaction force between the hand exoskeleton and the environment in the perpendicular direction to the attachment phalanx. Each finger is attached to the hand exoskeleton by means of a ring-like part placed on the medial phalanx, so this part is the main responsibility of the contact between the objects and hand exoskeleton system. In order to improve the grasping quality, each ring is covered by a non-slip material to increase the friction force with the objects.

### 2.4. Trunk-Pelvis Cable Driven Robot: Stand-Trainer

Detailed information regarding the robotic trunk-pelvis platform—stand trainer—can be found in Khan et al. [20]. The stand-trainer has a modular design and allows for the control of six degrees-of-freedom at the pelvis and three degrees-of-freedom at the trunk. The end effectors are belts that were strapped on the pelvis or mid-region of the trunk. The system can apply assistive, resistive, and perturbation forces that can be adjusted in real-time while using the software interface.

Controlled perturbations were delivered for the purpose of the present research. In order to execute these perturbation forces, we placed the four cables that were attached to the belt in a planar configuration, while using one cable along each diagonal. For forces in the anterior/posterior and lateral directions, the two neighboring cables are used in order to calculate the required tensions for achieving a desired Cartesian force. This force is added to the minimum cable tension to counter the effects of the force on the opposing cables. The applied force is trapezoidal with a rise time, constant time, and fall time for a desired force magnitude.

### 2.5. Acquired Data

The ground reaction forces and center of pressure were acquired by means of a force plate (Bertec, Columbus, OH, USA) and the reaction forces in the external support by means of a custom-made instrumented hand rail (±1000 N tri-axial, Bertec, Columbus, OH, USA); all of the forces are normalized as a fraction of the subject’s body weight. Kinematic data of the right upper-limb was acquired by placing infrared reflective markers in subjects’ wrist, elbow, and shoulder, as well as in the belt of the Trunk–Pelvis Robot. The position of this marker was tracked by a set of motion capture cameras (Vicon Motion Systems, Oxford, UK). Markers that are placed on the right upper-limb are used to estimate the angle of the elbow (θ) by computing the angle between the upper-arm and lower-arm vectors.

The surface electromyography (sEMG) measures (TeleMyo DTS, Noraxon, Scottsdale, AZ, USA) were taken for four muscle groups of the right upper limb: forearm flexors (flexor carpi radialis, palmaris longus, and flexor digitorum profundus), forearm extensors (extensor carpi ulnaris and extensor digitorum), and biceps and triceps brachialis. The signals were processed while using two different pipelines in order to obtain different outcomes. For obtaining amplitude-related information, signal was bandwidth filtered to remove high-frequency noise and motion artifacts (30–300 Hz) and smoothed by taking RMS value with a 500 ms time window; it was then normalized by dividing the signal by the maximum value that was measured for the corresponding muscle and subject along all of the conditions.

A hand exoskeleton recorded the interaction force between the finger modules that were equipped with force sensors (±20 N, 100 Hz, own fabrication [19]) and the hand rail. Force data were filtered while using a sixth order Butterworth low-pass filter with a cut-off frequency of 5 Hz, in order to remove electromagnetic noise and small vibrations that were introduced by the control loop of the actuators

### 2.6. Data Reduction

The postural performance is assessed while using the maximum displacement of the center of pressure (COP) of the subject. For this purpose, for each perturbation the initial COP position (average value for the 0.5 s previous to the beginning of the perturbation) has been substracted to the COP position for each instant during the perturbation, obtaining the displacement vector of the COP. The module of this displacement vector is computed for each time instant (ΔCOP) and the maximum ΔCOP value is obtained for each perturbation (ΔCOPmax). The characteristic performance value for a subject and condition is the average of the ΔCOPmax for all five perturbations.

Muscular performance was evaluated using three parameters that were extracted from the sEMG signals of each one of the four analyzed groups of muscles. In particular, the sEMG amplitude was normalized for each user and muscle by dividing the signal by the maximum value of all trials of its own user and muscle. Once normalized, for each trial the maximum sEMG amplitude (sEMGmax), integral of the signal (sEMGint), and time to maximum amplitude (t2max) were computed.

For a better understanding of the signal evolution, an epoch analysis of the sEMG signals was also performed. For this purpose, the trials were divided in 200 ms epochs and the average value of the sEMG values in each time window was taken as the representative value of the epoch. Therefore, visual or statistical analysis can be performed for certain time instants.

### 2.7. Statistical Analysis

The R software environment was used. The assumption of normal distribution was examined with the Shapiro–Wilk test and visually explored with normal Q-Q plots.

The assumption of data normality for exoskeleton interaction force and EMG-related variables was violated. For these parameters, the use of non-parametric testing was justified. Friedman test was used in order to detect significant differences across conditions. In case statistical differences were found, Wilcoxon signed-rank test was used as post-hoc analysis. The Holm–Bonferroni Method was used to adjust for family-wise error rate correction.

On the other hand, the COP results are shown to be normally distributed. In this case, One-way Repeated Measures ANOVA was employed. In order to evaluate whether the sphericity assumption has been violated, Mauchly’s test of sphericity has been used. If sphericity assumption is violated, using Greenhouse–Geisser correction when epsilon is ϵ≤0.75 and Huynh–Feldt correction when epsilon is ϵ>0.75 was employed. In case statistical differences were found, Bartlett’s test was used in order to study the assumption of equal variances across groups (homoscedasticity or homogeneity of variances). Based on the results of this test, Games–Howell Post-Hoc Test or Tukey post-hoc tests were used.

In all cases, *p*-values between the corrected *p*-value and 0.1 were only interpreted as a marginal significant difference for exploratory purposes.

## 3. Results

### 3.1. Subjects Functional Assessment

**Figure 3:**
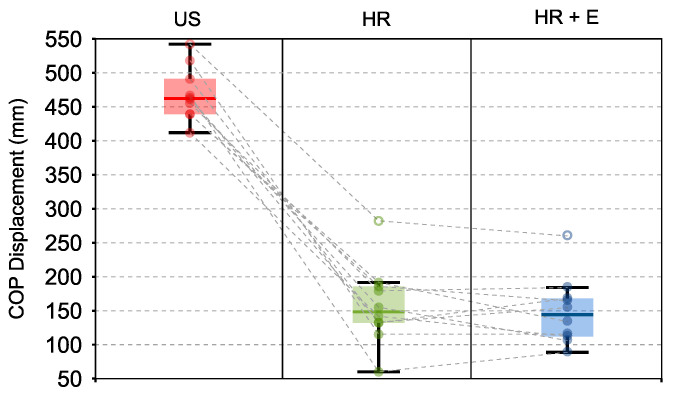
Average of the maximum COP displacement computed for each user along each of the three conditions: Unsupported (US), Hand rail support (HR), and Hand rail support with hand exoskeleton (HR + E). There is a significant reduction of this displacement when the external support is used, but there is not a significant difference between using the exoskeleton or not.

Figure 3 shows how there is a generalized reduction of COP displacement when the hand rail support is added (One-way Repeated Measure ANOVA p<0.0001). In fact, the displacement of the subjects’ COP during the condition HR + E is significantly lower than during the condition US (H1: Null hypothesis rejected, *p*≈0.00). However, when comparing conditions HR and HR + E, there is not a significant difference in the performance in terms of COP displacement (H2: Null hypothesis not rejected, *p*=0.72).

Further exploratory analysis of the functional parameters shows that the displacement of the markers in the belt that is attached to the subjects’ chest (One-way Repeated Measure ANOVA p=0.0084) does not present a significant difference between HR and HR + E conditions ([HR + E] − [HR] = 6.73 ± 24.24 mm; Post-Hoc; p=0.95). There is also no significant difference in the variation of the elbow angle (Δθ) between these two conditions ([HR + E] − [HR] = 2.04 ±3.97; Post-Hoc; p=0.16).

### 3.2. Upper-Limb Muscular Activity

**Figure 4:**
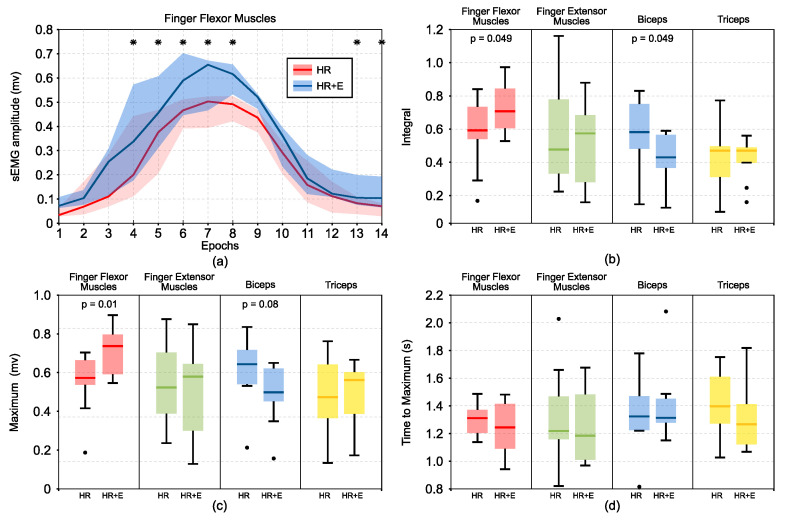
Graphical representation of the sEMG results. (**a**) Evolution of mean sEMG value during each condition. Shaded areas contains all of the mean values of all participants. Asterisk indicate a significant difference between conditions in that epoch. (**b**) Box plots comparison of the integral of the sEMG signal of each muscle in every condition.(**c**) Box plots comparison maximum sEMG activity of each muscle in every condition. (**d**) Box plots comparison of the time to maximum sEMG activity of each muscle in every condition. Outliers are shown as black dots.

Figure 4 show all of the results related with the Upper-Limb Muscular Activity. Results show that there is a significant difference in the integral of the variation (Figure 4b) of the sEMG in the finger flexor muscles (H3 [flexor]: Null hypothesis rejected, p=0.049) and in the Biceps (H3 [biceps]: Null hypothesis rejected, p=0.049). A Post-Hoc analysis of the maximum variation of the sEMG signal (Figure 4c) also points in the same direction, since there is a significant difference between conditions for finger flexor muscles (Post-Hoc: *p* =0.01) and a clear trend toward significance in the case of the Biceps (Post-Hoc: p=0.02).

In particular, finger flexor muscles show a higher activity when using the hand exoskeleton (sEMG integral =0.731±0.156) in comparison to the condition without exoskeleton (sEMG integral =0.578±0.21). This difference can also be observed in the finger flexor muscles evolution during the activity (Figure 4a). The time to maximum sEMG activity has been computed and no significant differences between HR and HR + E conditions has been found in any muscle.

The aforementioned muscular measures were computed as a difference with respect to the initial sEMG value at the beginning of the perturbation. The variation between the conditions of this initial value has also been explored and the difference between HR and HR + E conditions for finger flexor muscles has a certain trend toward significance (Post-Hoc: p=0.064). When the hand exoskeleton is used (HR + E), the initial sEMG value for flexor is greater (initial sEMG =0.056±0.022) than in the HR condition (initial sEMG =0.04±0.03).

### 3.3. Hand Exoskeleton Interaction with Hand Rail

**Figure 5:**
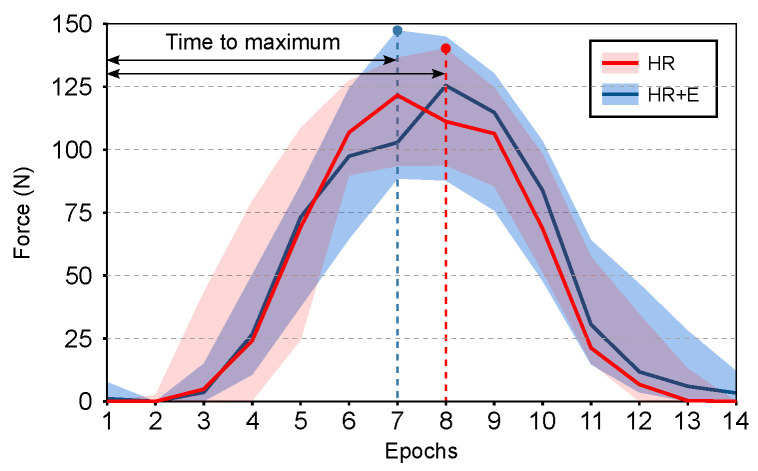
Evolution of the resultant force in the instrumented hand rail by epochs of 200 ms. Shaded areas contains all of the mean values of all participants in that condition.

In Figure 5, the evolution of the resultant force in the instrumented hand rail is shown in epochs of 200 ms. The results do not present a significant difference between HR and HR + E conditions ([HR + E]−[HR]=−1.13 ±14.94; Post-Hoc; p =0.88). However, the peak force is reached earlier when subjects wear the hand exoskeleton ([HR + E]−[HR] = 0.097 ± 0.13 s; Post-Hoc; p=0.064).

**Figure 6:**
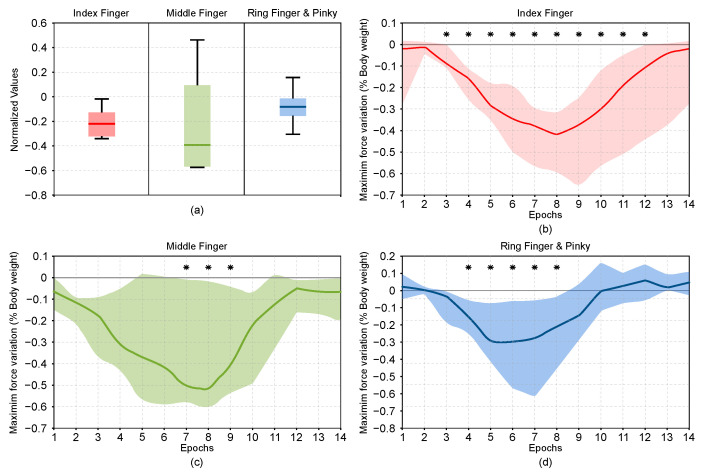
Results of the variation of the exoskeleton interaction forces. (**a**) Box plots of the exoskeleton interaction forces of all the subjects in every condition. (**b**) Evolution of interaction force of the index finger by epochs of 200 ms. (**c**) Evolution of interaction force of the middle finger by epochs of 200 ms. (**d**) Evolution of interaction force of the ring and pinky fingers by epochs of 200 ms. Shaded areas contains all the mean values of all participants. Asterisk indicates a significant difference from the reference of the force interaction in that epoch. In all graphs, positive values indicate a force that tries to open the finger modules of the hand exoskeleton. Negative values indicate a force that tries to close the finger modules of the hand exoskeleton.

Force sensors that are placed on the finger modules of the hand exoskeleton reveal that most of the subjects contract their hand, even if they were told not to do so, which results in a reduction of the total interaction force between the exoskeleton and the environment. Single sample *T*-test for each finger shows a significant reduction of the force for all fingers, as shown in Figure 6.

## 4. Discussion

The present study in able-bodied individuals undergoing controlled perturbations with the Stand-Trainer reveals that the hand exoskeleton did not interfere with their postural control performance, as we corroborated in H1 and H2. In both cases (HR and HR + E), the subjects improved their standing stability when compared to receiving postural perturbations without hand support, as measured by postural COP. This result seems to also be supported by the measurement of the displacement of the belt of the robot (torso).

The use of the hand exoskeleton does not result in a clear difference in terms of trunk postural control. When taking into account that subjects do not present major physical or psychomotor impairments, it should be assumed that they are able to maintain a good balance of the trunk on their own. The results suggest that the exoskeleton does not interfere in the trunk postural control of unimpaired people. Therefore, this suggests that it could present benefits for impaired people who are not able to grip properly. In addition, for post stroke patients, the use of this device in this type of application is a plus in motor training after the initial phases of rehabilitation after a stroke incident. From a rehabilitation point of view, in order to partially incorporate this device into daily living, activities could make a difference in the recovery of post stroke patients.

The presence of the hand exoskeleton seems to have an effect in the way the upper-limb is used to interact with the external support: this fact is suggested by the earlier peak time in the force of the bar when the hand exoskeleton is introduced. It also introduces differences in the activation of certain upper-limb muscles (Hypothesis 3). In particular, there is a significant and important increase in the muscular activation for flexor muscles. This is a generalized effect, since this increase is observed in the integral of the signal (time-sustained) and in the peak value (instant). This augmented activity might be associated with the fact that most of the subjects contract their hand, which results in a reduction of the interaction force that is measured in the hand exoskeleton. The extra effort that is exerted by the subjects is not applied in the bar, but mainly absorbed by the hand exoskeleton, which implements a rigid position control of the fingers. Muscular activity in the biceps presents some marginal differences between HR and HR + E conditions that, although they could be only spurious effects, are consistent with the other studied effects. In concrete terms, there is a marginal reduction of the integral of the sEMG signal.

Despite the encouraging results, a few study limitations should be discussed. We are aware that our study only involved eleven unimpaired subjects. Accordingly, while we believe that results can help us to know the effect of the use of the exoskeleton with impaired people, the results may not be generalized beyond the conditions of this study. The results should be investigated further, involving impaired people. Furthermore, this study has been limited only to the first reaction of the subjects to the hand exoskeleton, not allowing them time to learn new strategies. Therefore, the next logical step might be studying how the observed effects can be modified by the training in order to check whether subjects can make profit of the hand exoskeleton more efficiently by means of a constant training.

## 5. Conclusions

In summary, the utilization of the hand exoskeleton with the external support on non-impaired subjects seems to be as effective as using their fully capable hand from the functional point of view. However, despite there being no functional differences, the exoskeleton apparently introduces certain differences in the role of the upper-limb in their postural strategy. In the absence of further evidence, we hypothesize that these changes in the muscular activity could be associated to over-compensation strategies to try balance the lack of tactile and force feedback inherent to the utilization of a wearable device. This could explain the higher muscular activation previous to the perturbation, the application of unnecessary additional gripping force, and the anticipation in supporting on the handrail. In addition, the reduction in the time-sustained activation of the biceps and increase in the elbow maximum extension could denote that this lack of feedback could also make subjects slightly less confidence in their arm’s capabilities.

The exploratory nature of this study prevents us from making strong conclusions, but it allows for us to state more concrete questions regarding the use of a hand exoskeleton, not only as a tool to grasp and manipulate objects, but as a reliable interface to interact with the environment and the rest of the human neuro-muscular control system.

## Data Availability

Data available in a publickly accesible repository. The data presented in this study are openly available in FigShare at https://doi.org/10.6084/m9.figshare.13480272.v1 and https://doi.org/10.6084/m9.figshare.13480287.v1.

## Abbreviations

The following abbreviations are used in this manuscript:

PID	Proportional-Integral-Derivative
sEMG	Surface Electromyography
RMS	Root-mean square
COP	Center of pressure
nEMG	Normalized electromyography
